# Supplementary material for: High contrast holography through dual modulation
Source: Sci Rep. 2025 May 21;15:17615. doi: 10.1038/s41598-025-00459-8 (PMC12095464; doi:10.1038/s41598-025-00459-8)
Supplement: Supplementary file 1 — Supplementary Information. [file 41598_2025_459_MOESM1_ESM.pdf]

High contrast holography through dual modulation: Supplementary material

Leyla Kabuli[1,*], Oliver Cossairt[1], Florian Schiffers[1], Nathan Matsuda[1], Grace Kuo[1]

[1] Reality Labs Research, Meta, USA
[*] Corresponding author. Email: lakabuli@berkeley.edu

This supplementary material includes additional implementation details, simulation analysis, examples, and experimental results.

S1 Contrast error metric

Peak signal-to-noise ratio (PSNR) captures image quality, but does not specifically evaluate contrast. We developed a multi-level contrast metric to evaluate contrast performance.

As described in the main text, a common contrast metric is the Michelson contrast,

$$\mathscr{C}(\mathbf{I}) = \frac{\mathbf{I}_{\max} - \mathbf{I}_{\min}}{\mathbf{I}_{\max} + \mathbf{I}_{\min}}, \tag{S1}$$

which compares the highest and lowest intensities across an image. When the Michelson contrast is evaluated on a holographic image, speckle noise can give a false boost to the contrast value. Furthermore, evaluating the Michelson contrast on a full image does not capture variations in contrast based on spatial frequency.

In order to thoroughly evaluate contrast performance at multiple spatial frequencies, we represent the image as a Gaussian pyramid[1]. Each increasing Gaussian pyramid level captures lower-frequency image properties from low-pass filtering and downsampling operations. We compute Gaussian pyramids for both the target and the displayed hologram. At each Gaussian pyramid level, we compute the Michelson contrast for the target and the displayed hologram, and calculate the absolute error between target and hologram contrast. Finally, we sum the absolute error over all pyramid levels. We call this multi-level contrast metric "contrast error" (CE). An image with accurate contrast aims to minimize this quantity.

The equation to compute CE is

$$\text{CE}\left(\mathbf{I}, \hat{\mathbf{I}}\right) = \sum_{g} |\mathscr{C}(\mathscr{G}(\mathbf{I})) - \mathscr{C}(\mathscr{G}(\hat{\mathbf{I}}))|, \tag{S2}$$

in which the Michelson Contrast $\mathscr{C}(\cdot)$ is calculated on each Gaussian pyramid level g after applying the Gaussian pyramid $\mathscr{G}(\cdot)$ operator (low-pass filtering and downsampling). Here, $\mathbf{I}$ represents the target image and $\hat{\mathbf{I}}$ represents the displayed hologram. Better contrast images have lower CE.

Figure S1 shows experimentally captured images with both phase-only modulation and dual modulation ($\Delta_a = 480$ µm) and their corresponding multi-level contrast plots, which contain the Michelson contrast at each Gaussian pyramid level. Insets in the top left of each experimental capture show PSNR and CE. Pyramid level 0 corresponds to the full image and increasing pyramid levels correspond to further downsampled images. To compute CE with Eq. (S2), we sum the absolute error between the target and the hologram contrast across Gaussian pyramid levels.

S2 Analysis: modulator order and spacing

In the main text, we analyzed the effect of amplitude SLM pixel size on contrast and image quality. Other available design parameters are the ordering of the amplitude and phase SLMs and the spacing between the two SLMs. Here we study these parameters for three amplitude SLM pixel sizes ($\Delta_a = 64$ µm, 480 µm, and 2880 µm). The phase SLM pixel size is fixed at $\Delta_p = 8$ µm in this analysis.

First we consider the case where the amplitude SLM is placed before the phase SLM, and results are shown in Fig. S2a. In this configuration, the propagation distance after the phase SLM is fixed at 20 mm and the distance between the amplitude and phase SLM is swept between 0 and 10 mm. Metrics (PSNR and CE) are averaged over the set of grayscale images used in the main text. We see that when the amplitude SLM is first, the hologram quality does not depend on the distance between the two SLMs. This is the configuration that we demonstrated in experiment in the main text, with $\delta_z = 2.4$ mm.

Next, we consider the case where the phase SLM is placed before the amplitude SLM, and results are shown in Fig. S2b. Here, the total propagation distance, measured from the phase SLM to the image plane, is fixed at 20 mm. Then, the position of the amplitude SLM is swept across possible positions between the phase SLM and the image plane ($\delta_z = 0$ to 20 mm). In this configuration, improvement from dual modulation depends on the propagation distances, with better performance as the amplitude modulator is closer to the image plane. However, there is clear improvement at all distances tested compared to phase only. For short propagation distances between the SLMs ($\delta_z < \sim 5$ mm), the performance is similar regardless of which SLM is placed first.

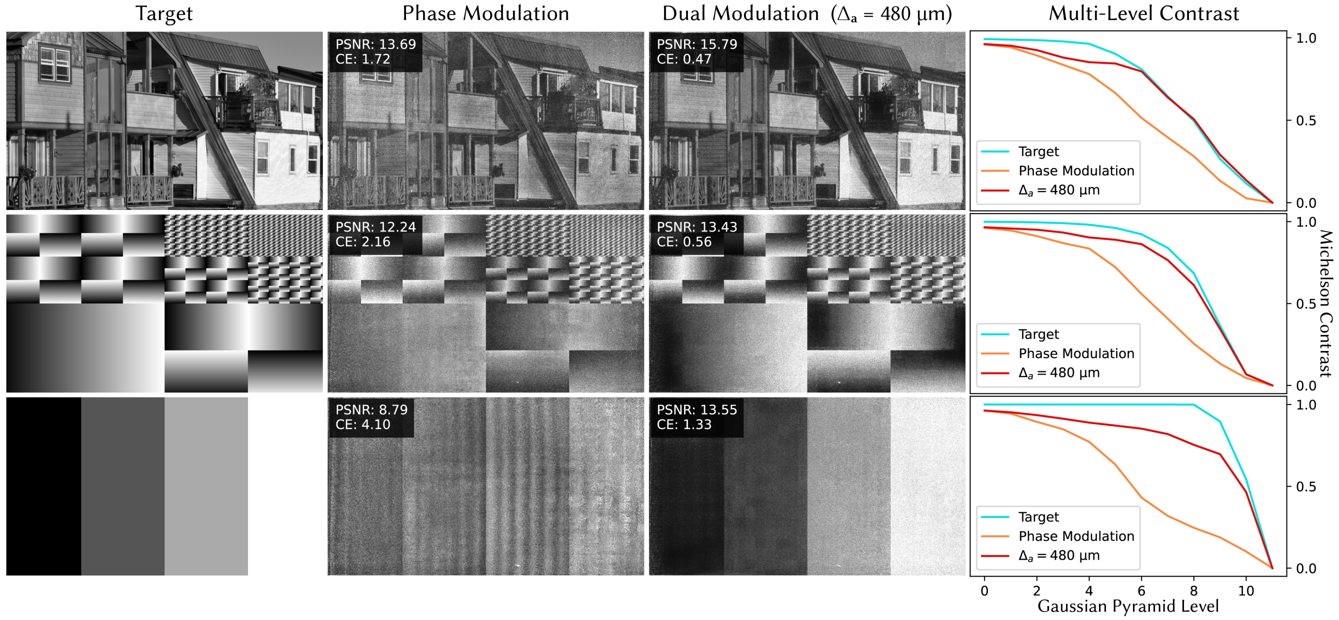

Figure S1. Contrast error (CE) metric. Experimentally captured images and their corresponding multi-level contrast plots. To form the contrast plots, the Michelson contrast is computed at all Gaussian pyramid levels for each experimental capture and target. Gaussian pyramid level 0 corresponds to the full image and increasing pyramid levels correspond to increasing downsampling. The absolute error between the contrast for the target and the captured image is summed across Gaussian pyramid levels to produce the CE value for each image. PSNR and CE are shown in the top left of each image. Houses source image by Madeleine Deaton (CC BY 2.0).

S2.1 Compact architectures

In the main text, we focused on the configuration with the amplitude SLM placed first, which was demonstrated with a benchtop experimental prototype. The alternative configuration that places the phase SLM first is well suited for compact architectures, with form factor compatible with near-eye displays. An example of such a compact architecture is visualized in Fig. S3. This design is based on the one proposed by Kim et al.[2], where a thin waveguide couples the illumination from a source to a reflective phase SLM. For dual modulation, a transmissive amplitude SLM can be added on the opposite side of the waveguide, closer to the eye. In this thin optical stack, light interacts with each SLM once, first with the phase SLM and then with the amplitude SLM. Our dual modulation approach may also be applied to other viable options for compact architectures, such as those involving multiple interactions with the amplitude SLM.

S3 Analysis: higher-order effects from amplitude modulator

Our experimental setup uses multiple $4f$ systems, which allow for filtering of the higher orders that arise from the pixel structure of the SLMs. However, as described in the main text, $4f$ systems are not compatible with a compact system, so it's advantageous if higher orders can be accounted for computationally instead of physically filtered. In particular, we propose using a transmissive amplitude modulator. Transmissive modulators generally have stronger higher-order effects due to their lower fill factor (compared to reflective modulators). Here, we show in simulation that given the large pixel pitches in dual modulation, fill factor is high and higher orders have negligible impact on image quality if the sub-pixel structure of the transmissive pixels is known.

Specifically, we simulate the case of reduced fill factor at the amplitude modulator. In a transmissive SLM, the electronics block light at each pixel, so we model the sub-pixel structure as an opaque, inactive border of fixed width around each pixel (see diagram in Fig. S4a). We assume that the inactive region is known in advance, and we take it into account during each iteration of optimization by multiplying the amplitude SLM pattern by a binary mask representing the inactive region. We simulate a range of amplitude pixel sizes from 32 μm to 960 μm while holding the inactive border width constant since the size of the electronics is independent of the pixel pitch. We consider three different border widths: 32 μm, 16 μm, and 0 μm, which corresponds to the case of perfect fill factor. In total, the simulation covers fill factors ranging from 25% to 100%.

We simulate both the configuration with the amplitude SLM first and with the phase SLM first. When the amplitude SLM is first, we simulate $\delta_z = 2.4$ mm between the amplitude SLM and phase SLM, followed by $z = 20$ mm propagation to the detector plane. When the phase SLM is first, we simulate $\delta_z = 4$ mm between the phase SLM and amplitude SLM with $z = 16$ mm propagation after the amplitude SLM to the detector plane. Note that the case with the amplitude modulator second could enable a compact architecture, as described in the main text. The simulated illumination is $\lambda = 520$ nm.

Fig. S4 shows the simulation results on both a natural image (a) and a sparse image (b). For the configuration with the amplitude SLM

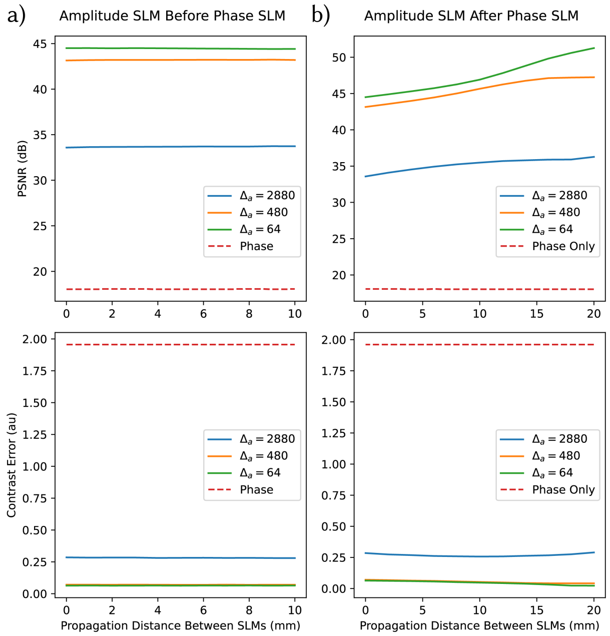

Figure S2. Dual modulation system configuration analysis. The ordering and spacing between the amplitude and phase spatial light modulator (SLM) affects PSNR and Contrast Error (CE). (**a**) With the amplitude SLM placed before the phase SLM, the spacing between the modulators does not affect metrics. (**b**) With the amplitude SLM placed after the phase SLM, performance depends on the spacing between the modulators. Regardless of the configuration, dual modulation provides significant improvement compared to phase only modulation. Metrics are averaged over the set of grayscale images used in the main text.

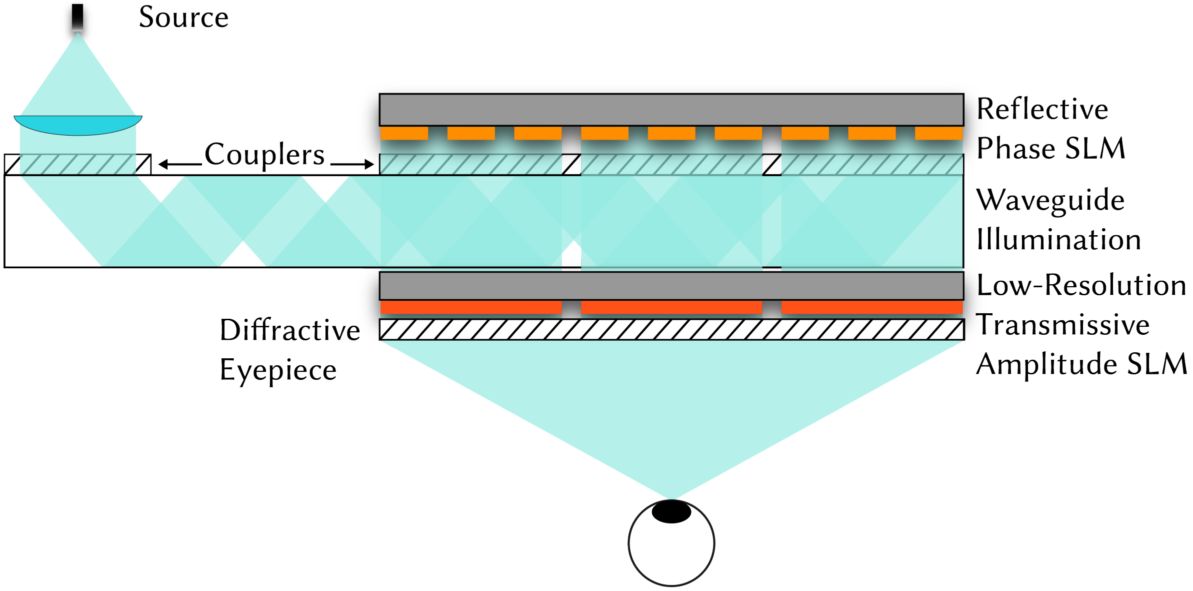

Figure S3. Compact architecture example. An example of a compact architecture for dual modulation places a reflective phase spatial light modulator (SLM) and a transmissive amplitude SLM on two sides of a thin waveguide, which couples the illumination from the source to the phase SLM. In this configuration light interacts first with the phase SLM and then with the amplitude SLM, in a thin form factor suitable for near-eye displays.

before the phase SLM (Fig. S4, left), higher-order effects are imperceptible across pixel sizes for both the natural and sparse image. There is a slight overall reduction in PSNR compared to the reference (0 µm border width) due to a portion of the incident illumination on the phase SLM being blocked by the amplitude SLM pixel structure. However, we see that PSNR remains high, even for larger inactive border widths

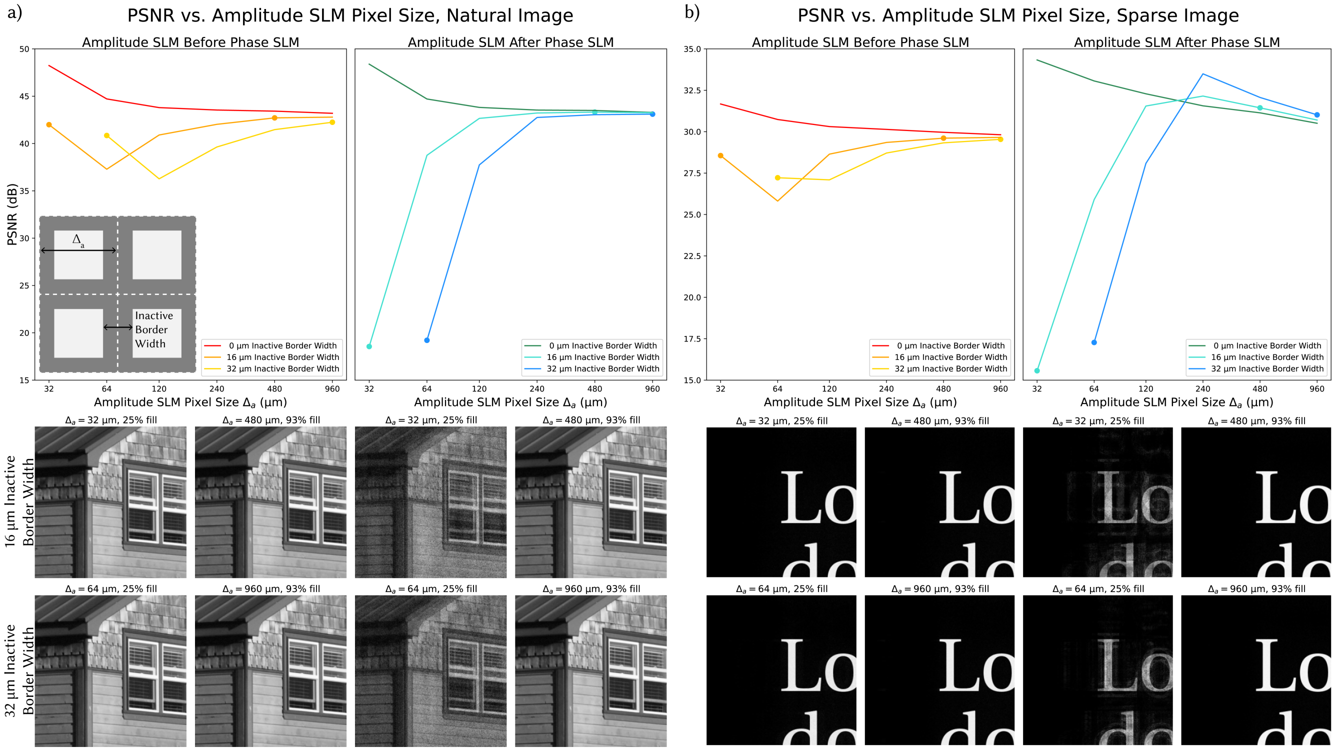

Figure S4. Amplitude spatial light modulator (SLM) fill factor. We analyze the impact of higher order effects from the amplitude SLM by simulating an inactive, opaque border around each amplitude pixel (depicted in the upper left inset). We compare several inactive border widths over a range of pixel sizes for both (**a**) a natural image of a house and (**b**) a sparse image of text. Note that border width of 0 μm corresponds to perfect fill factor, which will minimize higher orders. When the amplitude SLM is first, higher order effects can mostly be compensated for by the phase SLM, and there is minimal degradation from the incomplete fill factor. When the amplitude SLM is second, higher orders cause doubling in the image when the pixel size is small. However, for the large pixel pitches proposed in this work (∼480 μm), peak signal-to-noise ratio (PSNR) is high, and there is visually no doubling, even with a significant inactive border around each pixel. Houses source image by Madeline Deaton (CC BY 2.0).

(which correspond to smaller fill factor).

For the configuration with the amplitude SLM after the phase SLM (Fig. S4, right), smaller amplitude SLM pixel sizes ($\Delta_a = 32$ μm, $\Delta_a = 64$ μm) have visible higher orders due to the low fill factor. These higher orders are clearly visible as overlapping replicas in the sparse image of text. However, as amplitude SLM pixel size increases, image quality improves, and for large amplitude SLM pixel sizes ($\Delta_a = 480$ μm, $\Delta_a = 960$ μm), there are no visible higher orders and the PSNR is almost the same as the reference. In fact, with the sparse image, the unique combination of image content and pixel border structure can provides a slight boost in PSNR. As a reminder, in the main text we show that pixel pitches as large as $\Delta_a = 480$ μm are sufficient to generate high quality, high contrast imagery. Even assuming much more conservative border widths (16 - 32 μm) than the minimum suggested by Curatu and Harvey[3] (2.8 μm), we see that higher orders have negligible effect at these larger pixel sizes.

S4 Dual modulation light efficiency

As discussed in the main text, our approach involves attenuating incident light, as we combine a phase SLM with an amplitude SLM. A phase-only modulation approach is the most light-efficient, as it simply redirects light without attenuating. In contrast, a traditional LCD display is less light-efficient, as it produces images by attenuating light. Here, we show that our approach lands in between these approaches, as it is not only comparable to a traditional LCD display in light efficiency, but in fact more light-efficient for most image content.

For a grayscale image and corresponding amplitude pattern a normalized to the range $0-1$, we define its attenuation as $1-a$, where each pixel of the amplitude pattern is subtracted from 1. The average attenuation, or light attenuation, is taken across all pixels of the attenuation, and a lower light attenuation value, corresponding to higher light efficiency, is desirable.

We quantify the light efficiency of a traditional LCD display by considering a transmissive display where the image is formed solely by an amplitude pattern, where the amplitude pattern is the normalized image. For dual modulation, the light efficiency depends on the amplitude pattern resulting from the joint optimization of the phase and amplitude SLM patterns, also normalized. We can then compare the

Image	Transmissive Display	Dual Modulation ($\Delta_a = 64$ µm)	Dual Modulation ($\Delta_a = 480$ µm)	Dual Modulation ($\Delta_a = 2880$ µm
Street (Fig. 3)	0.669	0.500 / 25.23%	0.486 / 27.28%	0.654 / 2.09%
Stripes (Fig. 6)	0.500	0.506 / -1.13%	0.405 / 18.97%	0.420 / 15.97%
Ramp (Fig. 6)	0.500	0.454 / 9.35%	0.368 / 26.40%	0.412 / 17.57%
Gradient (Fig. 6)	0.500	0.468 / 6.54%	0.409 / 18.27%	0.453 / 9.37%
Star (Fig. 6)	0.609	0.432 / 28.96%	0.265 / 56.44%	0.540 / 11.33%
House (Fig. 6)	0.566	0.541 / 4.44%	0.514 / 9.18%	0.691 / -22.14%
Text (Fig. S4)	0.885	0.558 / 37.00%	0.512 / 42.21%	0.330 / 62.78%

Table S1. Light attenuation for traditional transmissive display vs. dual modulation. Average light attenuation for a traditional transmissive display and our dual modulation approach at three amplitude pixel sizes ($\Delta_a = 64$ µm, 480 µm, 2880 µm). 1 corresponds to full attenuation, 0 corresponds to no attenuation, and lower values correspond to better light efficiency. For each dual modulation pixel size we also include the percentage improvement in light efficiency over the transmissive display. Values are reported for each grayscale image used in this work. Dual modulation is up to 62.78% more efficient than a traditional transmissive display, and on average 19.34% more efficient.

light efficiency of these two approaches by calculating the light attenuation in each case.

Table S1 reports the light attenuation for both a traditional transmissive display and our dual modulation approach for the set of grayscale images used throughout this work. For dual modulation, we consider the three amplitude SLM pixel sizes ($\Delta_a = 64$ µm, 480 µm, 2880 µm) used in the main text. We additionally calculate the percent improvement in light efficiency of each dual modulation case relative to the traditional transmissive display. Dual modulation's light efficiency benefits are clear for sparse content (ex. Text, Star), high-frequency content (Street, Ramp), and low-frequency content (Gradient). For low-frequency content there is one case (Stripe) where dual modulation with $\Delta_a = 64$ µm is comparable to, but does not provide an improvement over a transmissive display, which we attribute to optimization noise. For the Stripe image, lower dual modulation resolutions maintain improvement in light efficiency. For high-frequency content there is one case (House) where the lowest resolution dual modulation with $\Delta_a = 2880$ µm, which is beyond the recommended bound for full light redirection of image content, is less light-efficient than a transmissive display. Overall, dual modulation is up to 62.78% more light-efficient and on average 19.34% more light-efficient than a traditional LCD display.

S5 Experimental prototype

A photograph of our benchtop experimental prototype is shown in Fig. S5. As described in the main text, we use two SLMs. A fiber-coupled laser diode ($\lambda = 520$ nm) is the illumination. Crossed polarizers convert the first SLM to modulate amplitude (SLM1). A $4f$ system relays the amplitude SLM to a short spacing $\delta_z = 2.4$ mm behind the phase SLM (SLM2). Another polarizer converts incident light to linear polarization for modulation by the phase SLM. A second $4f$ system relays the SLMs to the sensor plane. Apertures in the Fourier plane of each $4f$ system block higher orders.

S6 Calibration details

S6.1 Calibration: dual spatial light modulator (SLM) calibration and modeling

Achieving high-quality experimental results with a dual modulation system introduces additional challenges with system parameter calibration and SLM alignment and registration. To address these, we design a forward model for a dual SLM setup, adapting the approaches of Multisource Holography[4] and Neural Holography[5]. Our system model incorporates several components, including a source model (Sec. S6.1.1), propagation model (Sec. S6.1.2), phase and amplitude SLM models (Sec. S6.1.3), a dual SLM alignment model (Sec. S6.2), and a spatially varying aberration model (Sec. S6.3).

S6.1.1 Source model

The source model combines an incident plane wave with the non-idealities in the system (e.g. a slow varying intensity modulation in the source or dust/scratches in the optical path). We learn a complex-valued source pattern that corrects for these non-uniformities across the input plane wave.

S6.1.2 Propagation model

We use angular spectrum method (ASM) propagation[6] to model free-space light transport. To include global aberrations into the propagation model, we incorporate a learned complex pupil function.

S6.1.3 Phase and amplitude SLM models

Generating a hologram starts with the voltage values sent to the SLMs, represented by 8-bit digital input values. These values for the phase and amplitude SLMs are passed through a learned lookup table (LUT), mapping digital input to phase and amplitude for their respective SLMs.

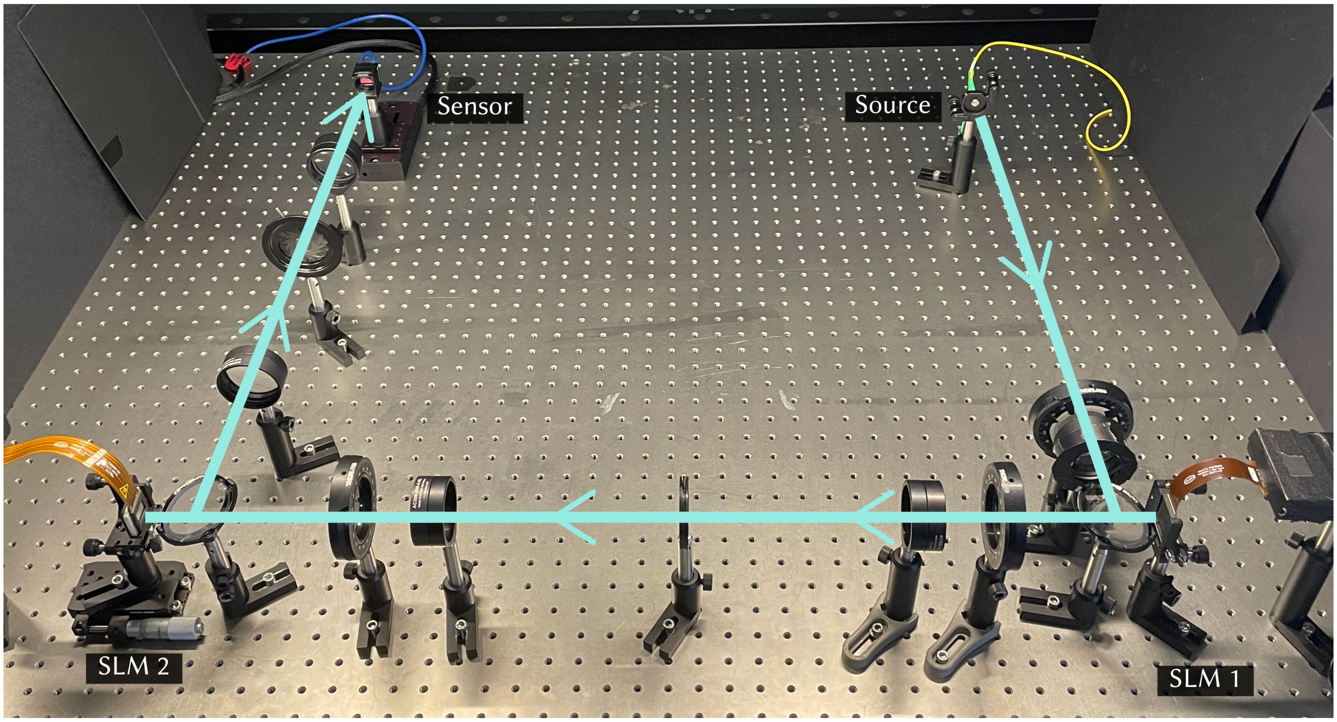

Figure S5. Benchtop experimental prototype. Photograph of benchtop experimental setup with optical path highlighted.

The phase LUT is parameterized by 256 coefficients corresponding to the 8-bit input values. The amplitude LUT uses a modification of the phase LUT parameterization. Amplitude modulation is periodic over the full actuation range of 256 coefficients. We select a contiguous region of 100 coefficients corresponding to one cycle of monotonic decreasing amplitude modulation from full transmission to full attenuation. For variable resolution amplitude modulation, we bin pixels by holding digital input values constant over all of the pixels in a binning region. Both LUTs are made differentiable by using 1D interpolation between coefficients.

Liquid-crystal-based SLMs have a characteristic known as field fringing (cross-talk), where actuation at a pixel affects neighboring pixels due to the transitions at pixel boundaries. This cross-talk between pixels is modeled with a learnable convolution kernel. Our model upsamples modulation values $2\times$ in horizontal (x) and vertical (y) directions to account for sub-pixel effects, with the cross-talk kernel being 5 pixels in upsampled space.

The phase with field fringing and the amplitude with field fringing are then converted to electric fields. For the amplitude modulation, we assume uniform phase, and for the phase modulation, we assume uniform amplitude. This approach allows us to model the amplitude modulation separately, providing a more accurate representation of the system.

S6.2 Calibration: mapping the complex field between modulators

Generating high-quality images with a dual spatial light modulator (SLM) system requires knowledge of pixel-level alignment between modulators. We achieve this by establishing a deformation map between the two SLMs, referred to as SLM1 and SLM2. This involves transforming the complex field from SLM1 into the coordinate system of SLM2. This transformation is done with the thin plate spline (TPS) model using the implementation provided by Kornia[7].

We first optimize a pattern for each SLM to display a grid of dots, which are displayed and captured sequentially on the camera. We use an asymmetric pattern of dots to account for orientation changes caused by the beamsplitter and the $4f$ systems. After identifying the centers of these dots within the camera's coordinate system, we fit a TPS model from SLM1 to the camera and another from SLM2 to the camera. We then apply linear algebra principles to calculate the TPS transformation between SLM1 and SLM2. We further refine the parameters of this transformation through gradient descent, along with other parameters within our computational model.

The transformation process between SLM1 and SLM2 is illustrated in Fig. S6. Due to minor alignment issues, we cannot use the full field of view of the SLMs. Therefore, our image quality evaluations are limited to areas where both SLM1 and SLM2 effectively modulate the wavefront.

S6.3 Calibration: modeling spatially varying aberrations

Spatially varying aberrations present a significant challenge in holography, as they can compromise the quality of the displayed images in 2D and 3D. These aberrations can result in significant disparities between the reconstructed images and their intended targets. Traditional models often assume a linear shift-invariant (LSI) system, with a consistent point spread function across the field of view (FOV). However, this assumption does not hold true for many optical systems, which have spatially varying point spread functions, necessitating a more complex

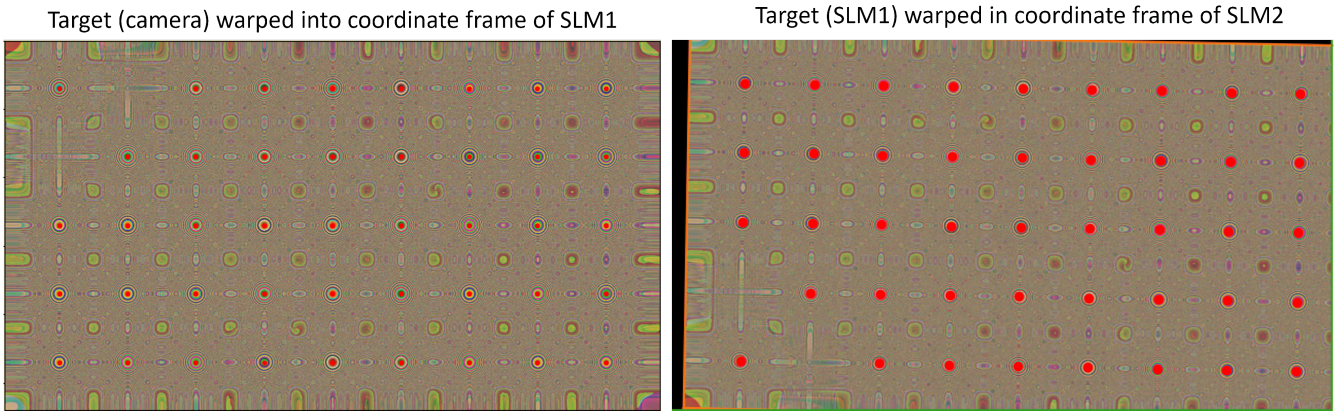

Figure S6. Spatial light modulator (SLM) and camera alignment. The left image shows a visualization of a grid of dots (asymmetric) reprojected into the coordinate system of the first spatial light modulator (SLM). The captured dots align with the pattern that was displayed on the first SLM (SLM1) to create these dots. The second image shows the Fresnel pattern after the transformation from the first SLM (SLM1) into the frame of the second SLM (SLM2). We also reproject the dots from the image capture back into the frame of the second SLM, which overlays well.

model. In our system we have two $4f$ relays and other system non-idealities (e.g. shifts in aperture placement in the Fourier plane) which cause spatially varying aberrations.

We tackle spatially varying aberrations in holographic displays by utilizing a patch-wise method. Each patch in the display incorporates a learned aperture function, allowing for localized aberration correction. This method enhances image quality by addressing aberrations at the local level in the FOV. We first patch the complex field into a grid of 18 x 24 of aforementioned aperture functions. Each spatial aberration function is represented by a 256×256 complex-valued function, which is resized to the resolution of the patch using bilinear interpolation in the complex domain using phase and amplitude respectively. We then perform patch-wise convolution for each patch using the corresponding aperture function in Fourier space. Afterwards we blend the patches together to model the effect of the spatially varying aberrations over the full FOV. In some experimental captures, the boundaries between aberration patches are visible. CITL is able to correct for most of the boundary effects that were not successfully blended.

As there are two $4f$ relays in our dual-SLM system, we learn separate spatially varying pupil functions for each relay. We also include a global learned aperture for each relay.

S6.4 Calibration: pipeline

S6.4.1 Calibration datasets

We create calibration images by first optimizing random phase holograms (with random phase initialization) from a dataset of natural images. We generate the phase SLM patterns by optimizing with an ideal ASM propagation model (Sec. S6.1.2). The primary advantage of using pre-optimized patterns is that we can see image features during the software calibration. An alternative would be to use speckle patterns where random phase is displayed on the hologram as proposed in Multisource Holography[4]. This method is expected to work equally well, but the absence of any interpretable image features makes it challenging to debug the calibration process for phase SLMs. For the amplitude SLM we use a combination of low-frequency patterns (e.g. constant, large blocks, and stripes) and high-frequency patterns (e.g. speckle patterns).

S6.4.2 Calibration procedure

To learn the parameters of our dual SLM model, we use a three-step calibration procedure with gradient descent in PyTorch. For each calibration step, we capture an experimental dataset of images corresponding to input SLM patterns.

We first learn the parameters corresponding to the phase SLM using a dataset of 330 pre-computed random phase patterns. The amplitude SLM is held constant in this dataset, and during training, only parameters corresponding to the phase SLM are updated.

We then learn the parameters corresponding to the amplitude SLM using a dataset of 500 patterns, consisting of a combination of low-frequency and high-frequency patterns. Similarly, the phase SLM is held constant in this dataset and only parameters corresponding to the amplitude SLM are updated during training.

Finally, we fine-tune all the parameters for the dual system, using a dataset of 50 pre-computed random phase and smooth amplitude patterns. The dual system patterns are optimized with the calibrated model with individually learned amplitude and phase components.

The three-step training process reaches convergence in approximately 8 hours on an Nvidia A6000 GPU. All implementation of our calibration was done using HoloTorch[8].

S6.4.3 Hologram generation

After training our dual SLM model, we solve for the SLM patterns for amplitude and phase for each desired hologram. The patterns are initialized with constant (smooth) amplitude for the amplitude SLM and random phase for the phase SLM. Patterns are optimized with the

constraint of being in the monotonic region for the amplitude SLM and phase wrapped for the phase SLM. A dual pattern optimization for a 2D hologram is on the order of minutes on an Nvidia A6000 GPU.

To vary the resolution of the amplitude SLM, we initialize patterns at desired resolutions, then use a differentiable upsampling operation to make constant blocks formed from pixels at the same resolution as the phase SLM before propagating through the model. In back-propagation, the learnable parameters in the amplitude SLM are the initial low resolution values.

S7 Active camera-in-the-loop (CITL)

Active camera-in-the-loop (CITL)[5] is an online calibration process that we use to fine-tune images, correcting for small model mismatch and artifacts. CITL adjusts the SLM patterns for one hologram based on live feedback from the camera.

We first optimize the amplitude and phase SLM patterns offline based on our learned calibration model (Sec. S6). Displaying these two patterns and capturing the resulting hologram gives our starting point for CITL. We use the transformation process of our TPS model (Sec. S6.2) to transform the camera capture to the SLM space to do our optimization. This transformation gives us precise pixel-to-pixel alignment between the captured image and target, which allows for replacing the simulated model output used in our training loop with the transformed camera capture. We update the amplitude and phase SLM patterns for the hologram based on back-propagation with the transformed camera capture at each gradient descent step. Fig. S7 demonstrates the results of fine-tuning. CITL image quality improvement is clearly visible, as it reduces speckle, image artifacts, and improves color fidelity.

S8 Lowest resolution modulation

At the lowest resolution amplitude modulation, where amplitude pixel size is $\Delta_a = 2880$ µm, individual pixel boundaries are visible in experimental captures. This resolution is beyond the recommended bound in the main text, so light cannot be fully redirected within the amplitude pixel region by the phase SLM. In Fig. S8, the pixel boundaries are visible in both experimental captures and experimental captures with CITL fine-tuning. CITL corrects slightly for some of the pixel boundary artifacts but cannot suppress all of the amplitude pixel structure. For images with high-frequency content, such as the Mondrian building, the pixel structure is less visible in experimental captures. The simulated outputs produced by our calibrated model do not contain any perceptible pixel boundaries. This suggests that with improvements to the calibration approach, it may be possible to further correct for the mismatch between experimental captures and simulated model outputs. Future work can move towards using extremely low resolution amplitude modulators with reduced pixel boundary artifacts.

S9 Amplitude patterns

Here we include examples of the amplitude SLM patterns for both 2D images and focal stacks.

Fig. S9 demonstrates amplitude patterns for the grayscale simulated images in the main text, for amplitude pixel sizes $\Delta_a = 64$ µm, $\Delta_a = 480$ µm, and $\Delta_a = 2880$ µm. Full light transmission corresponds to 1.0 and full light attenuation corresponds to 0. The amplitude pattern resembles the low-frequency structure of the target.

Fig. S10 demonstrates amplitude patterns for the focal stacks in the main text, with amplitude pixel size $\Delta_a = 480$ µm. The left pattern is for the green channel of the natural scene color focal stack and the right pattern is for the grayscale focal stack containing text and a Siemens star. Full light transmission corresponds to 1.0 and full light attenuation corresponds to 0. Intuitively, the amplitude pattern resembles the low-frequency structure of the target, attenuating light in dark regions and transmitting light in bright regions. As the intensities of objects do not vary significantly through defocus, one amplitude pattern captures the structure across the focal stack.

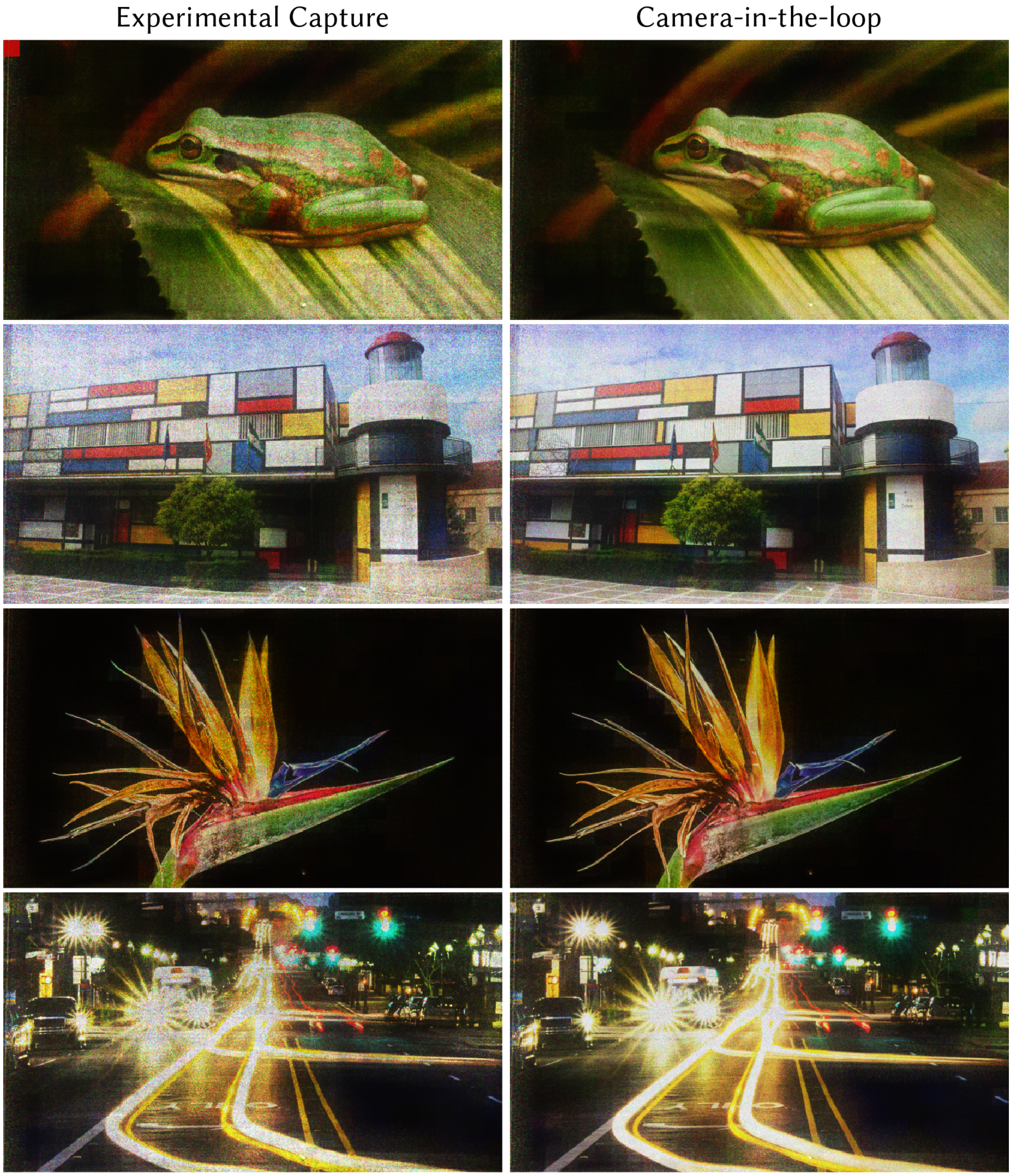

Figure S7. Active camera-in-the-loop (CITL) comparison. In the first column we show experimentally captured holograms optimized with our learned model but no active CITL. In the second column we show experimentally captured holograms that were iteratively fine-tuned based on camera feedback using CITL. Amplitude pixel size is $\Delta_a = 480$ µm, shown in red in the top left image. Flower source image by Paul Longinidis (CC BY 2.0).

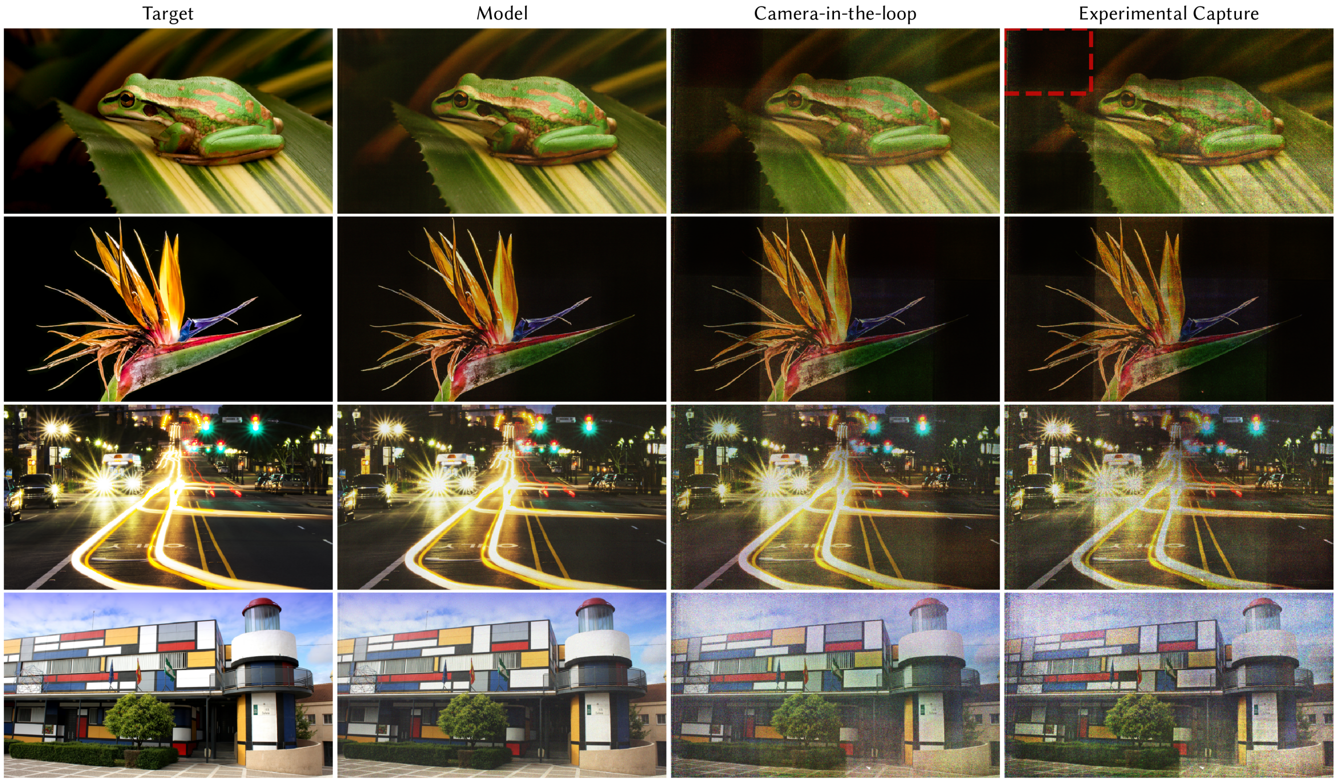

Figure S8. Lowest resolution dual modulation. At very low resolution amplitude modulation (amplitude pixel size $\Delta_a = 2880$ µm), light is not fully redirected within the amplitude pixel region by the phase modulator, resulting in visible pixel boundaries in the experimental captures of holograms optimized with our learned model and the experimental captures with additional camera-in-the-loop (CITL) fine-tuning. The calibrated model output shows that the pixel boundaries are imperceptible in simulation, indicating potential for improvement in experimental suppression of pixel boundaries. Amplitude pixel size is shown in red in the top right image. Flower source image by Paul Longinidis (CC BY 2.0).

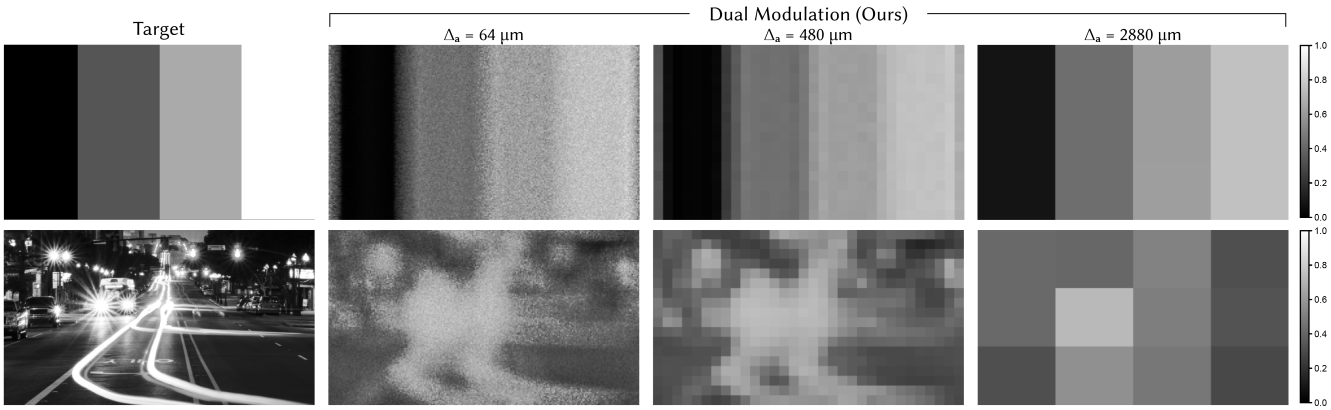

Figure S9. 2D image amplitude patterns. Amplitude patterns for amplitude pixel sizes $\Delta_a = 64$ µm, $\Delta_a = 480$ µm, and $\Delta_a = 2880$ µm. Patterns correspond to the simulation grayscale images in the main text. Full light transmission corresponds to 1.0 and full light attenuation corresponds to 0. The amplitude pattern resembles the low-frequency structure of the target.

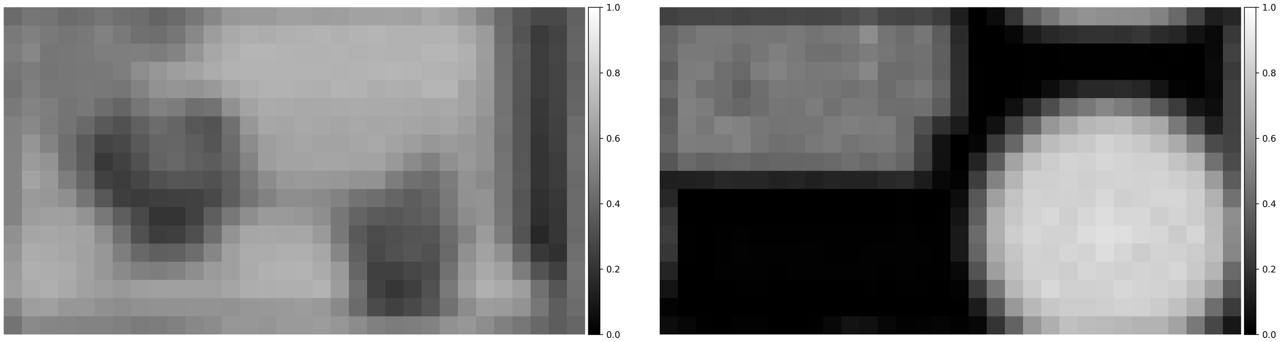

Figure S10. Focal stack amplitude patterns. Amplitude patterns for amplitude pixel size $\Delta_a = 480$ µm. Patterns are for the natural scene color focal stack (left) and the grayscale focal stack (right) from the main text. Full light transmission corresponds to 1.0 and full light attenuation corresponds to 0. In general, the amplitude pattern resembles the low-frequency structure of the target.

Acknowledgments

Flower source image by Paul Longinidis (CC BY 2.0) is available at https://www.flickr.com/photos/128235612@N06/18033751041/. Image was cropped but otherwise unmodified. Houses source image by Madeleine Deaton (CC BY 2.0).

References

1. Burt, P. & Adelson, E. The laplacian pyramid as a compact image code. *IEEE Transactions on Commun.* **31**, 532–540 (1983).

2. Kim, J. *et al.* Holographic glasses for virtual reality. In *ACM SIGGRAPH 2022 Conference Proceedings*, SIGGRAPH '22 (Association for Computing Machinery, New York, NY, USA, 2022).

3. Curatu, G. & Harvey, J. E. Analysis and design of wide-angle foveated optical systems based on transmissive liquid crystal spatial light modulators. *Opt. Eng.* **48**, 043001–043001 (2009).

4. Kuo, G., Schiffers, F., Lanman, D., Cossairt, O. & Matsuda, N. Multisource holography. *ACM Trans. Graph.* **42** (2023).

5. Peng, Y., Choi, S., Padmanaban, N. & Wetzstein, G. Neural holography with camera-in-the-loop training. *ACM Transactions on Graph. (TOG)* **39**, 1–14 (2020).

6. Goodman, J. W. *Introduction to Fourier optics* (Roberts and Company publishers, 2005).

7. Riba, E., Mishkin, D., Ponsa, D., Rublee, E. & Bradski, G. Kornia: an open source differentiable computer vision library for pytorch. In *2020 IEEE Winter Conference on Applications of Computer Vision (WACV)*, 3663–3672 (2020).

8. Chakravarthula, P. *et al.* Differentiable cameras and displays. In *ACM SIGGRAPH 2022 Courses*, 1–213 (2022).
